# Supplementary material for: Microencapsulation of a novel Bacteroides thetaiotaomicron strain: a promising strategy to fortify intestinal barrier fortification in a weaned pig model
Source: Appl Environ Microbiol. 2026 Jul 27;92(8):e00503-26. doi: 10.1128/aem.00503-26 (PMC13488332; doi:10.1128/aem.00503-26)
Supplement: Supplementary file 1 [file aem.00503-26-s0001.docx]

**Supplementary Materials**

**Fig. S1** Bacteriostatic test of LYH5 bacterial fluid. (A) LYH5 co-cultured with *E. coli* ATCC 25922; (B) LYH5 co-cultured with *S. cholerae suis* ATCC 527; (C) LYH5 co-cultured with *S. aureus* ATCC 25923.

**Fig. S2** Morphology of sediment obtained by ultracentrifugation under scanning electron microscope.

**Fig. S3** Results of single factor test of LYH5 microcapsule. (A) Embedding rate with different concentrations of Sa; (B) Embedding rate with different concentrations of gellan gum; (C) Embedding rate with different bacteria - gum ratios ; (D) Embedding rate with different concentrations of CaCl₂. Different lowercase letters above the bars represent significant differences with *P* < 0.05.

**Fig. S4** LYH5 microcapsule freeze-dried.

**Table S1 Dietary formulation and nutrient level (air dry basis, %)**

| Item (Ingredient) | Content | Item (Nutritional level) | Content |
| --- | --- | --- | --- |
| Corn (7.8% crude protein) | 28.60 | ME, (Mcal kg-1) | 3.35 |
| Extruded corn (7.8% crude protein) | 31.05 | Crude protein | 17.60 |
| Soybean meal (44.2% crude protein) | 6.98 | Crude fibre | 2.04 |
| Extruded soybean | 8.46 | Calcium | 0.35 |
| Whey powder (low protein) | 4.66 | Available phosphorus | 0.35 |
| Fish meal (62.5% crude protein) | 3.71 | Lysine | 1.23 |
| Soybean protein concentrate | 5.99 | Methionine | 0.41 |
| Sucrose | 1.90 | Methionine + Cysteine | 0.67 |
| Soybean oil | 1.54 | Threonine | 0.72 |
| Limestone | 0.60 | Tryptophan | 0.20 |
| Dicalcium phosphate | 0.43 |  |  |
| L-Lysine·HCl (78%) | 0.30 |  |  |
| NaCl | 0.19 |  |  |
| Chloride choline | 0.14 |  |  |
| DL-Methionine | 0.11 |  |  |
| L-Threonine (98.5%) | 0.02 |  |  |
| Tryptophan (98%) | 0.01 |  |  |
| Vitamin premix^1^ | 0.05 |  |  |
| Mineral premix^2^ | 0.29 |  |  |
| Total | 100.00 |  |  |

^1^ Vitamin premix supplied per kilogram of diets: 15000 IU vitamin A, 4000 IU vitamin D_3_, 40 IU vitamin E, 5 mg vitamin K_3_, 2.5 mg biotin, 2.5 mg folic acid, 25 mg pantothenic, 5 mg thiamine, 3.6 mg vitamin B_6_, 600 μg vitamin B_12_.

^2^ Mineral premix per kilogram of basic feed: 100 mg Fe (FeSO_4_·H_2_O),6 mg Cu (CuSO_4_·5H_2_O),4 mg Mn (MnSO_4_·H_2_O),100 mg Zn (ZnSO_4_·7H_2_O),0.30 mg I (KI) and 0.35 mg Se (Na_2_SeO_3_.)

**Table S2 Physiological and biochemical identification results of B. thetaiotaomicron LYH5**

| Item | Result |
| --- | --- |
| Catalase test | Negative |
| Gelatin liquefaction test | Negative |
| Exercise test | Negative |

**Table S3 Antibiotic resistance test results of LYH5**

| Antibiotics | Code of drug sensitive paper | Valence (μg/tablet) | Criteria for determining the diameter of IZD | | | IZD/mm | Resistance |
| --- | --- | --- | --- | --- | --- | --- | --- |
|  |  |  | R | I | S |  |  |
| Amoxicillin | AML | 10 | ≤13 | 14～17 | ≥18 | 18 | S |
| Enrofloxacin | ENR | 5 | ≤27 | 28～36 | ≥37 | 27 | R |
| Sulfadiazine | SUZ | 300 | ≤12 | 13～16 | ≥17 | 30 | S |
| Streptomycin | SUZ | 10 | ≤11 | 12～14 | ≥15 | 0 | R |
| Rifampin | RD | 5 | ≤16 | 17～19 | ≥20 | 38 | S |
| Polymyxin B | PB | 300 | / | / | ≥11 | 11 | S |
| Tetracycline | TE | 30 | ≤14 | 15～18 | ≥19 | 48 | S |
| Clindamycin | DA | 2 | ≤14 | 15～20 | ≥21 | 22 | S |
| Ampicillin | SAM | 20 | ≤11 | 12～14 | ≥15 | 38 | S |
| Linezolid | LZD | 30 | ≤20 | 21～22 | ≥23 | 40 | S |
| Cefepime | FEP | 30 | ≤14 | 15～17 | ≥19 | 0 | R |
| Imipenem | IPM | 10 | ≤13 | 14～15 | ≥16 | 44 | S |
| Clarithromycin | CLR | 15 | ≤13 | 14～17 | ≥18 | 0 | R |
| Vancomycin | VA | 30 | ≤14 | 15～16 | ≥17 | 11 | R |
| Penicillin G | PB | 10 | ≤28 | - | ≥29 | 15 | R |
| Gentamicin | CN | 10 | ≤12 | 13～14 | ≥15 | 0 | R |

R: Drug resistance; I: intermediary; S: Sensitive.

**Table S4 Analysis of variance for response surface model of LYH5 microcapsule encapsulation effiencey**

| Source | Sum of Squares | df | Mean Square | F Value | P-value |
| --- | --- | --- | --- | --- | --- |
| Model | 1027.65 | 14 | 73.40 | 30.65 | < 0.001 |
| A- SA (%) | 9.89 | 1 | 9.89 | 4.13 | 0.062 |
| B- Gellan glue (%) | 82.39 | 1 | 82.39 | 34.40 | < 0.001 |
| C- CaCl2 (%) | 331.99 | 1 | 331.99 | 138.62 | < 0.001 |
| D- Bacteria-gum ratio | 0.04 | 1 | 0.04 | 0.02 | 0.894 |
| AB | 4.29 | 1 | 4.29 | 1.79 | 0.202 |
| AC | 2.25 | 1 | 2.25 | 0.94 | 0.349 |
| AD | 0.12 | 1 | 0.12 | 0.05 | 0.827 |
| BC | 6.28 | 1 | 6.28 | 2.62 | 0.128 |
| BD | 0.51 | 1 | 0.51 | 0.21 | 0.6518 |
| CD | 0.27 | 1 | 0.27 | 0.11 | 0.7448 |
| A^2 | 466.96 | 1 | 466.96 | 194.97 | < 0.001 |
| B^2 | 190.01 | 1 | 190.01 | 79.33 | < 0.001 |
| C^2 | 81.32 | 1 | 81.32 | 33.95 | < 0.001 |
| D^2 | 91.21 | 1 | 91.21 | 38.08 | < 0.001 |
| Residual | 33.53 | 14 | 2.40 |  |  |
| Lack of Fit | 27.00 | 10 | 2.70 | 1.65 | 0.332 |
| Pure Error | 6.53 | 4 | 1.63 |  |  |
| Cor Total | 1061.18 | 28 |  |  |  |

**Table S5. Comparison of apparent nutrient digestibilities of pigs in different groups**

|  | Group | | | | *P-*value |
| --- | --- | --- | --- | --- | --- |
| Item | CON | T | TJ | J |  |
| Energy | 83.10±0.30 | 82.97±0.39 | 83.25±0.49 | 82.99±0.54 | 0.965 |
| DM | 81.86±0.50 | 81.96±0.41 | 81.75±0.50 | 81.88±0.55 | 0.991 |
| CP | 72.87±0.75 | 74.94±0.69 | 72.18±0.90 | 71.77±1.29 | 0.093 |
| EE | 69.35±0.71 | 67.50±0.74 | 69.68±0.95 | 69.35±0.90 | 0.221 |
| CF | 20.92±2.53 | 23.95±1.61 | 21.19±2.38 | 21.59±1.91 | 0.758 |

CON: control group; T: basal diet +LYH5 live bacteria; TJ group: basal diet +LYH5 microcapsule; J group: basal diet + empty capsule. Data are presented as mean ± SEM, values with different superscript letters within the same row indicate significant differences (*P* < 0.05).

**Table S6. Comparison of blood biochemical indices of piglets in different groups**

|  | Group | | | | SEM | *P-*value |
| --- | --- | --- | --- | --- | --- | --- |
| Item | CON | T | TJ | J |  |  |
| TP(g/L) | 54.34 | 57.16 | 57.35 | 53.30 | 2.90 | 0.249 |
| ALB(g/L) | 28.83 | 29.74 | 30.36 | 27.95 | 2.00 | 0.650 |
| GLO(g/L) | 25.52 | 24.84 | 27.00 | 25.35 | 2.15 | 0.775 |
| ALP(U/L) | 172.38 | 197.50 | 195.25 | 174.50 | 17.14 | 0.326 |
| ALT(U/L) | 51.88 | 49.61 | 57.76 | 48.11 | 6.22 | 0.439 |
| GLU(mmol/L) | 5.89 | 6.24 | 5.50 | 5.58 | 0.66 | 0.675 |
| TG(mmol/L) | 0.44 | 0.47 | 0.52 | 0.46 | 0.64 | 0.589 |
| UREA(mmol/L) | 3.65 | 2.96 | 3.23 | 3.38 | 0.40 | 0.393 |
| NEFA(μmol/L) | 233.84 | 196.29 | 376.66 | 205.41 | 75.69 | 0.083 |

CON: control group; T: basal diet +LYH5 live bacteria; TJ group: basal diet +LYH5 microcapsule; J group: basal diet + empty capsule. TP: Total Protein, ALB: Albumin, GLO: Globulin, ALP: Alkaline Phosphatase, ALT: Alanine Aminotransferase, GLU: Glucose, TG: Triglyceride, UREA: Urea, NEFA: Non-Esterified Fatty Acid. Data are presented as mean ± SEM, values with different superscript letters within the same row indicate significant differences (*P* < 0.05).
